# Empowering Mayo Clinic Individualized Medicine with Genomic Data Warehousing

**DOI:** 10.3390/jpm7030007

**Published:** 2017-08-22

**Authors:** Iain Horton, Yaxiong Lin, Gay Reed, Mathieu Wiepert, Steven Hart

**Affiliations:** Mayo Clinic, Rochester, MN, 55905, USA; horton.iain@mayo.edu (I.H.); lin.yaxiong@mayo.edu (Y.L.); reed.gay@mayo.edu (G.R.); wiepert.mathieu@mayo.edu (M.W.)

**Keywords:** personalized medicine, precision medicine, next-generation sequencing, NGS, genomic variant call format, gVCF, pharmacogenomics, translational research

## Abstract

Individualized medicine enables better diagnoses and treatment decisions for patients and promotes research in understanding the molecular underpinnings of disease. Linking individual patient’s genomic and molecular information with their clinical phenotypes is crucial to these efforts. To address this need, the Center for Individualized Medicine at Mayo Clinic has implemented a genomic data warehouse and a workflow management system to bring data from institutional electronic health records and genomic sequencing data from both clinical and research bioinformatics sources into the warehouse. The system is the foundation for Mayo Clinic to build a suite of tools and interfaces to support various clinical and research use cases. The genomic data warehouse is positioned to play a key role in enhancing the research capabilities and advancing individualized patient care at Mayo Clinic.

## 1. Introduction

Recent advances in genomics and other molecular technologies have ushered in the era of individualized medicine (also known as personalized or precision medicine), in which each individual’s genetic makeup can provide insight into the diagnosis and prognosis of disease and can help to predict the response to treatment. Individualized medicine is a priority at the Mayo Clinic, a nonprofit organization with major campuses in Rochester, MN, USA; Scottsdale and Phoenix, AZ, USA; and Jacksonville, FL, USA and an associated Health System with dozens of locations in Minnesota, Wisconsin, and Iowa, USA. The Mayo Clinic is organized around the three “shields” of clinical practice, education, and research, with strong integration among these three areas. This integration provides the opportunity to quickly translate cutting-edge research into clinical practice, but also requires infrastructure that meets both clinical and research needs.

The Center for Individualized Medicine (CIM) is one of several major centers at the Mayo Clinic. Its mission is to discover and integrate the latest in genomic, molecular, and clinical sciences into personalized care for each Mayo Clinic patient. It consists of five Translational Programs (Biomarker Discovery, Clinomics, Epigenomics, Microbiome, and Pharmacogenomics) and seven Infrastructure Programs (Bioethics, Bioinformatics, Biorepositories, Education, Administration, Information Technology, and Medical Genome Facility). The Individualized Medicine Clinic (IM Clinic) enables scientists and physicians to integrate whole-genome and whole-exome sequencing into patient care. Three consulting services are offered: a Cancer service line, a Diagnostic Odyssey service line, and a Pharmacogenomics (PGx) service. These services contribute to clinical care by, respectively, identifying drug targets in tumors for the treatment of advanced cancer; enabling genetic diagnoses that inform treatment plans; and inserting drug alerts based on patients’ genomic profiles into their electronic health records (EHRs), ensuring that prescribed medications are both appropriate and delivered at the right dose.

After the Human Genome Project was completed in 2003, CIM was created with the goal of sequencing the genome for every patient. The ability to store these genomic results and relate them to clinical phenotypes, along with the ability to translate research discoveries to clinical practice, was seen as a necessary step to bring individualized medicine to our patients. However, in the early days of CIM, many different databases and applications were in use across Mayo Clinic, preventing efficient synthesis and application of this information. 

In 2008, CIM began to integrate genomic data with the Mayo Enterprise Data Trust [1] (now UDP—Unified Data Platform) through a Mayo-IBM (IBM Corporation, Armonk, NY, USA) collaboration, which produced the Biologically Oriented Repository Architecture (BORA). BORA focused on gene expression, methylation, and single-nucleotide-polymorphism (SNP) data generated from array-based sequencing technologies. Although BORA provided a centralized repository and the ability to execute queries based on clinical phenotype and genomic attributes, the data was gathered from a variety of sources which may adopt different experimental designs and data processing workflows, making integration and comparison across studies difficult. 

These limitations, along with the advent of next-generation sequencing (NGS), led to the need for more sophisticated and scalable architecture. In 2011, major funding from Mayo Clinic and its benefactors allowed for expansion of CIM into an organization as it exists today. The purpose of this article is to describe how Mayo Clinic’s CIM has implemented a genomic data warehouse to manage this integration of genomic and clinical data, including the technical and organizational approaches, challenges, and key decision points.

## 2. Implementation of the Warehouse

### 2.1. Genomic Data Warehouse

**Figure 1:**
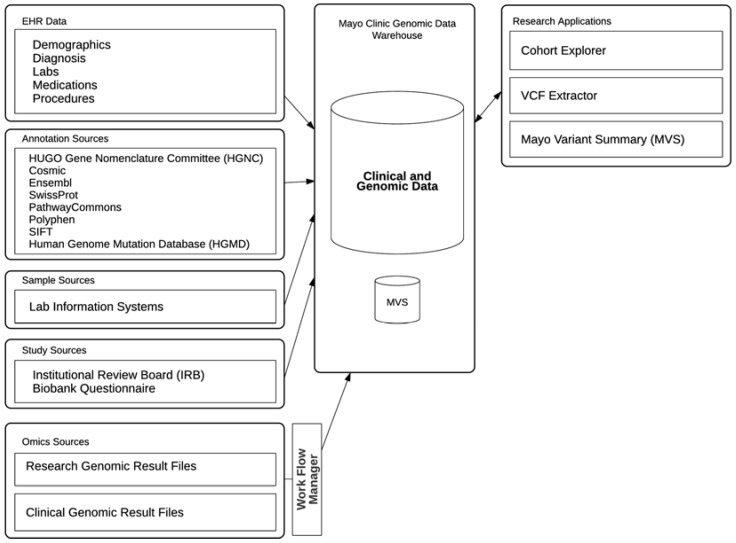
Mayo Clinic genomic data warehouse architecture. EHR: electronic health records VCF: Variant Call Format2.2. Project Timeline

The Mayo Clinic genomic data warehouse system is built on the Oracle Translational Research Center (Oracle TRC) product, which provides a central repository, an Oracle database, to store clinical data and genomic results mapped to various genomic annotations as shown in Figure 1. Cohorts of patients or research subjects can be built using Structured Query Language (SQL) or through Cohort Explorer (CE), a web-based application included with the Oracle TRC product suite. A suite of applications has been built around this genomic data warehouse (referred as TRC from herein) to meet various needs in the clinical practice and genomic research at Mayo Clinic.

**Figure 2:**
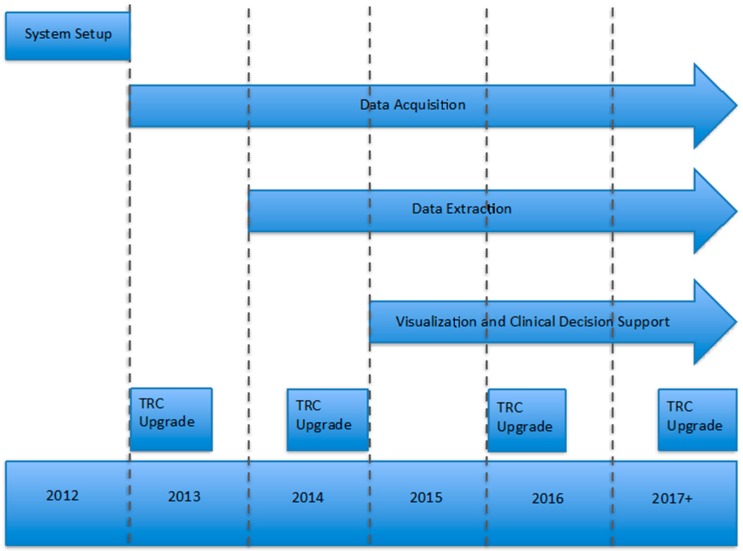
Mayo Clinic genomic data warehouse implementation timeline. TRC: Translational Research Center.

TRC was first implemented at Mayo in 2012 (Figure 2); acquisition and installation of hardware and software and initial training took approximately four months. The focus for 2013 and 2014 was to build, automate, and refine data pipelines to create the diverse dataset of genomic results and associated clinical phenotype data. Beginning in 2015, we began developing applications around TRC to gain insight from our repository of genomic variants and test out new capabilities of clinical decision support in areas like pharmacogenomics. The plan for 2017 includes product updates to extend our analytical capabilities through the application programming interfaces (APIs), upon which new and existing applications can be easily integrated to support existing and emerging clinical and research workflows.

Major releases of the TRC product typically follow a yearly schedule while patches are delivered as needed when high priority issues are identified and fixed by the vendor. As with any vended product, the customer must consider product release schedules and plan internal efforts involving the IT system accordingly. Major releases of TRC typically require at least two months of effort for installation, configuration, and validation across our development, integration, and production environments.

### 2.2. System Setup

The installation team consisted of a cross-functional group with members of different skills and backgrounds: technical, scientific, project management, and executive oversight expertise (Table 1). Orchestrating the procurement and installation of this platform involved more than 23 Mayo Clinic staff. 

Although the setup of TRC was owned and executed by the information technology (IT) organization, collaboration and orchestration across multiple areas within Mayo Clinic were necessary to implement this system. The stakeholders across our research and clinical organizations were instrumental in its success. For example, Bioinformaticians and Statisticians provided scientific support and implemented software to integrate the bioinformatics pipelines into TRC. The CIM Oversight Committee, which consists of administrators, program directors and stakeholders from various areas, provided strategic guidance for these collaborative efforts and for project prioritization. A Scientific Oversight committee was created to consult with the IT (information technology) team on technical decisions involving implementation of genomic data and to align our efforts with the needs of genomic research and clinical service lines. With members from Bioinformatics, Biostatistics, and IT, this committee has continued throughout the entire project lifecycle and remains a critical aspect of our success.

### 2.3. Hardware and Reference Data Specifications

The hardware acquired to run this system in our Production environment is a combination of Oracle Exadata Database Machine, Oracle ZFS Storage Appliance (ZFS) for backups and file staging, and two servers for hosting Oracle Weblogic Server (Table 2). Using Oracle Exadata Database Machine infrastructure has allowed us to capitalize on its Hybrid Columnar Compression feature that offers a lower storage footprint due to achieving 10x compression and better performance because of 10x reduction in disk scan I/O. Sizing the database hardware was most dependent on projecting the amount of genomic result data to load over the expected useful life of the hardware. The clinical phenotype data is much smaller in size and has significantly less impact on the hardware required to power TRC. Sizing the application hardware for Cohort Explorer and other web based tooling was more difficult because we needed to estimate a user base that did not exist as of implementation time.

A defined set of publically available reference data is supported in TRC, complete with prebuilt loaders (Table 3). Using this reference data, we can correlate our stored variants with gene nomenclature records, function prediction, disease, etc. The main challenge in housing this data within TRC is keeping it current. We currently review and refresh these data sets on an ad hoc basis. 

### 2.4. Data Acquisition and Loading

After the installation was completed, a subset of the original setup team (Table 4) began to develop the Extract-Transform-Load (ETL) and data pipeline process to populate the genomic warehouse with clinical data and existing genomic results. The key challenge in this effort was dealing with diverse and non-standard data formats. 

The vended ETL tool, Oracle Data Integrator (ODI), was used to acquire, transform and load the discrete data elements from the Mayo Clinic UDP and Lab Information Management systems (LIMS) into TRC. The presence of the Mayo Clinic UDP was a significant benefit to our efforts. The UDP is an institutional data warehouse that normalizes and consolidates data from our various EHR systems. In addition to having the benefit of a consolidated data store to retrieve clinical phenotype information, we also benefited from the expertise of IT staff within this area to complete the mapping and automation of ETL processes to populate the clinical phenotype components of TRC. Most importantly, this isolation of our system from the existing EHR implementation provides protection of impact from upstream changes to EHR systems. As of mid-2017, Mayo Clinic has begun an institution wide effort to replace our existing EHR with Epic. Having the UDP as our clinical phenotype source means that corresponding changes to our software due to EHR replacement should remain minimal.

**Figure 3:**
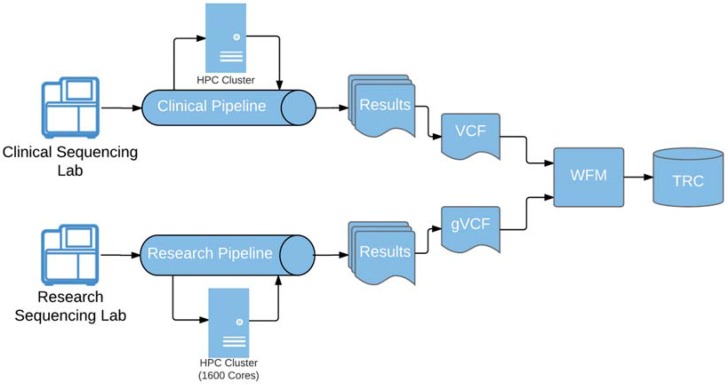
Automated result pipeline and process. Fully automated genomic data validation and load process is managed by Workflow Manager (WFM). Data files in Variant Calling Format (VCF) and genomic VCF (gVCF) are dropped off in a specific folder on the high-performance computing (HPC) cluster where WFM automatically picks up the files, processes and loads them into TRC.

Loading of genomic results was significantly more challenging. The genomic result file load utility provided by the vendor was manual, error-prone and most importantly lacked required data validation. The development of a custom-built automated load workflow management (WFM) system was necessary to perform the required data validation, alleviate the operational burden as well as streamline the processing of the genomic result data as it is delivered from clinical and research bioinformatics pipelines (Figure 3). 

Built on Apache Camel, an Apache open-source integration framework, WFM allows support staff to effectively monitor and handle exceptions during the entire workflow process starting at the file drop-off location through successfully loaded data into TRC. The entire genomic data ingestion process is managed through this software and equips our staff with the ability to monitor and troubleshoot problems that can occur within any of the validation or loading steps that occur. It also gives us control over throttling the flow of result data into the system and gives control to the support staff at multiple checkpoints in the data ingestion process. 

### 2.5. Support for Multiple Types of Genomic Data

#### 2.5.1. Next-Generation Sequencing and Arrays

Today, NGS has become the dominant sequencing method in genomic research, and has quickly entered clinical practice [2]. Although array-based techniques will continue to exist, NGS has much higher fidelity and gives a more detailed representation of the genome [3], analogous to the generational increase in computer image resolution. This increased fidelity will enable greater discovery, in part by allowing reanalysis of genomic data as new analytic methods are introduced. TRC is well designed to store and query NGS data with corresponding clinical phenotype data. Storing and providing NGS data has high potential impact because knowledge about molecular underpinnings of disease, treatment, and heredity continues to advance at a rapid pace. This knowledge can be applied as it changes to existing results without having to acquire a new specimen and repeat testing. 

#### 2.5.2. Variant Call Format and Genomic Variant Call Format

Variant data—information about specific genomic locations that differ among individuals or from a human reference genome—are recorded in the Variant Call Format (VCF) [4]. VCF files are relatively small (with a small number of rows) because the difference in the DNA of any two individuals is less than 0.1% [5]. As a result, most of the nucleotide positions assayed/tested (but where no variations were observed (also called non-variant)) are not recorded in VCF format. In contrast, genomic VCF (gVCF) [6] files record data on all assayed positions, regardless if a variation was detected or not. The need to know the genotype information at those non-variant positions is critical to many of the research and clinical use cases described in Section 3 of this paper.

There are two major technical consequences to the decision to store gVCF data: increased cost of storage and decreased query performance. Storing gVCF in TRC consumes substantially more database space. A typical single-sample whole-exome VCF file (variant only) contains approximately 5000 to 20,000 rows consuming roughly 1 MB of database space; in contrast, a typical single-sample whole-exome gVCF (variant and non-variant) file contains 3 to 5 million rows and can take up to 45 MB of database space. 

A concern about gVCF is that the increased footprint will also cause degradation of query performance. To mitigate this concern, we have the ability to add additional hardware, to configure our bioinformatics pipelines to effectively compress non-variant data, or both, while minimizing data loss and not compromising fidelity. The compression is achieved by representing larger groups of non-variant records as single logical records through mechanisms available in the variant processing pipeline software. Despite these additional steps, the decision to store gVCF has proven to be crucial to support several uses cases as described later in this paper. Careful consideration is recommended for decisions on whether to store VCF or gVCF. We found our desire to implement solutions that utilized the same as reference information outweighed the cost of storing gVCF for our research studies. If storing non-variant results from genetic tests would not have perceived value, the recommended approach is to store VCF because of the additional scale and better performance in loading and querying data.

#### 2.5.3. Other Data Types

TRC is designed to support multiple genomic data types, such as variants, gene expression data, structural variants, and copy number variation, obtained from both array-based and NGS experiments. It allows users to query across these data types and clinical data to find cohorts of subjects meeting criteria of interest.

Phase 1 of TRC supports DNA sequencing data on SNPs, insertions/deletions (INDELs), and non-variant nucleotides. The structural variants, copy number variations, RNA variants, and sequencing data for other species will be supported as required in future phase. 

Clinical data types supported by TRC data model include: patient demographics, procedures, diagnosis, medications, diagnostic tests (Lab), and encounters. Although Mayo is converging 3 EHRs into a single EHR (Epic), historical data must be retrieved from many different EMRs and in-house developed systems within Mayo. There have also been scenarios where certain data elements are not loaded into TRC due to lack of end-user demand. Although we work to incorporate new data types as we encounter them, the time and resources to do so are not always immediately available.

Acquiring new genomic data types or results from existing data pipelines and loading into TRC is still a complex and time-consuming activity for our IT team. Much of the challenge involves taking data formatted in something other than VCF/gVCF and modifying it to conform to the VCF specification (VCF 4.1) that is required by TRC genomic result loaders. Careful attention must be given to how existing genomic pipelines produce data and what format the output is in. Output in VCF or gVCF format would be considered typical, but any deviation from that could add significant IT time and complexity during implementation.

#### 2.5.4. Support Whole Genome

The cost of whole-genome sequencing continues to decrease. The database space required to store whole-genome sequencing data will multiply by at least 50 times as whole-exome sequencing only covers ~2% of the entire genome. Such drastic increases in data volume will require substantial change to the core database architecture and design. Until a time when the value of having whole-genome result sets outweighs the cost of sequencing and storing results, we anticipate continued focus on whole-exome sequencing and targeted panels, particularly in the clinical space.

### 2.6. Security

Patient privacy has always been and will continue to be a requirement that cannot be compromised. The warehouse stores clinical and research data in the same database, which adds complexity to the data security implementation. Oracle Virtual Private Database (VPD) is used to control the access to the data. VPD provides fine-grained access control at the database level to protect discrete data element by obfuscating data or completely denying access to the data based on user’s credential and privileges. 

TRC is integrated with the internal Institutional Review Board (IRB) system to determine end user authorization for accessing information. Clinical phenotype data for Mayo Clinic patients is stored under a pseudo IRB and our study subject information is organized under IRB IDs specific to each study. IRB membership is loaded into our system from our IRB repository and is used to construct our security and access policies for VPD. Clinicians can be added to the pseudo clinical IRB for access to all patient data and clinically ordered genomic results. Users associated with research studies are automatically granted authority to access all data associated with IRBs they are assigned to in our source system. IRB membership can have a slightly different affect depending on how the data is being accessed. Data export directly from the warehouse requires IRB membership while direct query of the warehouse or data access through Cohort Explorer will contain obfuscated PHI information when a user is not a member of the IRB associated with the data. This allows users to answer feasibility questions and work with our IRB area to request access to specific studies when full non-obfuscated access is needed. 

Security and protecting patient privacy is a top-level concern of Mayo Clinic. Intellectual property could be exposed through data queried from TRC and must be protected from unauthorized use. Working closely with our IRB team and various leaders in our research organization helped ensure this concern was addressed early and in an acceptable way. 

### 2.7. Summary 

TRC provides the schema to effectively store at scale a very complex dataset along with solutions for loading genomic results. The selection of TRC allowed us to focus our efforts on integrating source data almost immediately with a technology platform that was supportable within our IT organization. The majority of our IT effort to date has involved loading various types of data into the system and working through highly complex TRC upgrades. Implementing a system of this scope requires domain expertise across the institution. Having a cross-functional team representative of those areas involved or impacted is key to success.

## 3. Benefits, Use Cases and Future Directions

TRC provides clinicians and researchers with the ability to gain insight across phenotypic and genomic data from diverse populations of patients and subjects. Clinicians evaluating a specific patient can easily find data from other patients with the same disease or symptoms and who have a similar phenotype and genotype. These similar cases will allow the clinicians to evaluate different treatment options, predict likely patient outcomes, and select the best treatment for their patients. For patients seen in the Mayo Clinic IM Clinic [7], the genomic data warehouse should prove to be an invaluable source of information for resolving diagnostic odysseys, treating cancers with targetable mutations, identifying potential adverse drug interactions, and more. 

In this section, we will describe some of these use cases.

### 3.1. Use Cases

#### 3.1.1. Biobank

More than 50,000 patients have been enrolled into a biospecimen repository, the Mayo Clinic Biobank. This resource includes data from diverse subjects and a rich collection of metadata that provides a valuable pool of study candidates for future genetic research. This program was instituted to support a wide array of research studies; because the Biobank is not disease-specific, it is particularly well suited to provide control samples for population-based studies [8]. 

The ability to identify cohorts of subjects whose genomes have already been sequenced can substantially save investigators the time and effort that otherwise would be required to recruit new subjects and sequence their genomes; this resource also facilitates the determination of study feasibility. The Biobank staff has the ability to find subjects with target genetic variations in combination with clinical phenotypes using Cohort Explorer (CE). The study team can use this information to plan the study design. The Biobank staff was trained on CE in June 2015.

#### 3.1.2. Data Reuse

With this central genomic warehouse, data gathered for previous studies can be repurposed for new studies and validation of existing studies. The researchers can easily identify a cohort of subjects of interest for a new study using CE and extract relevant genomic data into VCF files using the VCF Extraction application. By pooling the genomic data from multiple studies together, researchers can greatly enhance the statistical power for their studies and can conduct studies otherwise not possible due to difficulty in recruiting research subjects and costs in sequencing the samples. By leveraging the genomic data from existing studies, the life and value of the genomic results can be extended far beyond the boundary of the original studies that created them. As a result, TRC can reduce study startup time from weeks or months to, in some cases, less than a day. Focusing on marketing data reusability of TRC can drive adoption and interest from potential users of the system within an organization. Even though costs have been falling for years, the cost of genetic testing is still expensive and not normally reimbursed by health insurance.

#### 3.1.3. Platform for Hypothesis Generation

The availability of linked clinical and genomic data, including subject phenotypes, allows researchers and clinicians to quickly subject hypotheses to preliminary tests, or to generate new hypotheses from the existing data for potential new studies and funding proposals. For example, analyzing variant frequencies across our dataset and correlating this with clinical phenotypes can bring to light potentially causal relationships between rare or not-well-studied genomic variants and disease. This will equip researchers with the ability to discover and better understand the molecular underpinnings of disease.

### 3.2. Foundation to Build Additional Clinical Genomic Applications

#### 3.2.1. Mayo Variant Summary

**Figure 4:**
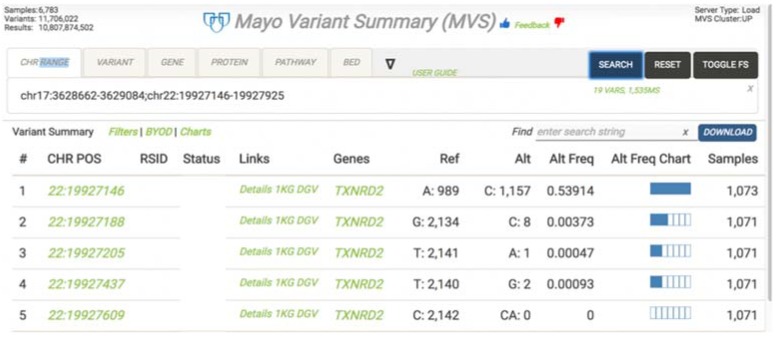
Mayo variant summary (MVS).

To support variant frequency based research at Mayo, the Mayo Variant Summary (MVS) application was developed to dynamically visualize variant frequencies across all samples stored in the warehouse (Figure 4). MVS is a highly scalable, cloud-native in-memory database solution that can summarize billions of variants and non-variant data across our entire genetic and phenotypic data set. As an example, this system can produce variant frequency results across a given chromosomal range and group results by gender or set of supplied demographics in 200 ms–1.5 s on average. 

MVS stores a pre-defined set of clinical attributes and sample metadata associated with the genomic results. It allows users to build a patient cohort by filtering on the clinical attributes and sample meta-data and retrieve the summarization of the variants on the selected cohort quickly. Clinical users can use MVS to assess how rare a variant is within the specified patient population while research users can use MVS to perform allele frequency analysis for various subject groups and population.

This use case was made possible by the decision to store gVCF result data, which enables the determination of variant frequency in a population. 

#### 3.2.2. Pharmacogenomics: Right Study

To be clinically useful, pharmacogenomics information must be delivered to the point of care at the right time—when medication orders are being entered. Mayo Clinic has developed a proof-of-concept solution, Molecular Decision Support, using TRC as the source of genomic data for a proprietary pharmacogenomics system. 

The service-oriented architecture of Molecular Decision Support provides ease of integration with other clinical information systems that assess patients’ molecular phenotypes and produce actionable information to physicians at the point of care through clinical decision support modules within our EHR. The RIGHT Study [9] is building the necessary infrastructure to trigger alerts to physicians during the drug ordering process so that patients get the right drug at the right time with the right dosage. 

This study will determine the best path forward for bringing a sustainable pharmacogenomics solution to the clinical practice using NGS data and EHR integration. Again, this use case is made possible by storing gVCF results data, which provides the required genotype alleles on both variant and non-variant positions to allow the molecular interpretation engine to assess the patient’s molecular phenotype.

#### 3.2.3. Variant Extraction

**Figure 5:**
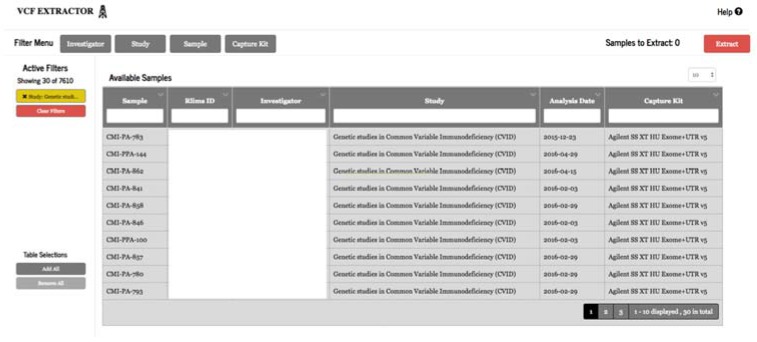
VCF extractor.

Researchers and clinicians often have their own favorite tools to study and analyze variant data. Variant Extraction, a web-based application, was developed to allow users to securely filter and extract genomic variant results from TRC for a specific cohort into VCF format and annotate the data with industry-standard annotation sources using our high-performance computing (HPC) cluster (Figure 5). Study IRB membership is required to extract genomic data. 

Extraction and annotation in this system is a two-step process. This application can extract variant call level information for 1000 samples and produce a VCF file ready to annotate in less than 20 min. The VCF file is then automatically sent to our HPC cluster for annotation that can be completed and delivered to the user in under an hour. In order to produce a VCF file for samples from different studies, both variant and non-variant data must be available for all samples to be included in VCF file on the positions where the variant was observed on any of samples. Once again, this use case is made possible with the decision to store gVCF results in TRC. We found it necessary to build a solution for VCF extraction because the mechanism delivered as part of TRC had limitations. Doing so gave us an opportunity to integrate data export with our genomic annotation platform that enables us to annotate our variants with internally and externally curated datasets. 

### 3.3. Data Statistics 

Since 2013 we have been able to load a significant amount of genomic data into the warehouse (Table 5). Automating load processes in order to take VCF and GVCF files directly from genomic data pipelines to the warehouse has allowed focus our efforts on bringing forwared solutions to serve our research needs and advance individualized patient care. 

### 3.4. Summary

Implementation and operation of a system of this scale is a significant undertaking. Gathering and fully understanding potential use cases within our organization proved the potential value of this solution and justified the investment. Post implementation IT effort to bring additional solutions forward to research and clinical areas is necessary to realize the full potential of this or similar platform. 

## 4. Challenges and Lessons Learned

TRC was successfully deployed to users at Mayo in 2013; however, as described below, the implementation has been challenging for a variety of reasons detailed below. 

### 4.1. Need to Include End Users Early in the Implementation Process

Identifying the end users of the system and involving them in the development of TRC was critical in shaping early decisions, such as the implementation of gVCF to meet the needs of our research community and clinical practice. However, we have discovered that additional outreach and communication with end users (principal investigators) would have been helpful both for shaping the system itself and for ensuring its use throughout the institution. For example, some users were not initially prepared to adopt the tools that were introduced because of a perceived lack of need or less-intuitive user interface. This pushed us to develop additional, potentially more user-friendly applications to meet user needs, as described in Section 3.2.

#### 4.1.1. Policies for Data Sharing and Security

Genomic results are unique to each patient and therefore can potentially be used to identify the patient, so the system architecture must include security and privacy controls. Access to genomic results must be managed and controlled to meet Healthcare Insurance Portability and Accountability Act (HIPAA) privacy requirements [10] and requirements of IRB designed to ensure that study-specific genomic results are not used without appropriate approval.

IRB plays a significant role in the warehouse security and privacy controls as it relates to access to information for our users. All data as it can be retrieved is associated with an IRB ID and this attribute is used to control what and how much information can be seen or exported. 

On the research side, investigators may be reluctant to share genomic results and even the study collected clinical data during the early stages of research for competitive reasons. To achieve researcher buy-in, discussions should be held early to ensure that everyone is comfortable with the security provisions and the proposed visibility of their genomic data. 

#### 4.1.2. Communication about the Pace of Innovation

Implementing TRC was a strategic decision by CIM driven by the desire of Mayo Clinic to be a leader in individualized medicine. Our focus during the first few years has been on data acquisition. Collaboration with Bioinformaticians and Statisticians have been crucial for integrating our bioinformatics pipelines with TRC to load genomic results. Demand for the data within the organization was originally low but has been growing steadily. 

This steady development of capability must be balanced with the fact that the fields of genomics and bioinformatics are changing rapidly. For example, the software libraries used in the bioinformatics pipelines change often and at different times. There is high demand in research to implement newer versions of these libraries that contain iterative advancements in algorithms to push the science forward, but these advancements can affect the output that we load into TRC and cause downstream problems in our genomic result workflows. To mitigate the impact of these changes to the system, we continue to focus on improving intra-organizational communication regarding software change management.

Mayo Clinic has made a significant investment both in implementing TRC and in ongoing operational requirements needed to keep pace with the rate of change in these areas. However, given the scope of change, the high demand to implement newer software or iterative improvements in existing software, and the multiple competing priorities of our IT organization, it has been imperative to be able to effectively manage expectations and priorities and to collaborate across organizational boundaries in order to meet the needs of our stakeholders. The collaboration with Oracle has been particularly important to our ability to implement and operationalize TRC while continuing to change and evolve along with the science. Partnering with a vendor may not always be feasible, but at the time we embarked on implementation, we felt it necessary in order to influence the product to fit our needs and vision. We feel this partnership has been productive in bringing the TRC product forward and improving it for other current and potential users.

#### 4.1.3. Outreach and Awareness

Organizational outreach and awareness is crucial to adoption, but within the large Mayo Clinic ecosystem of available software and infrastructure solutions, keeping users informed of the existence, capability, and change lifecycles of TRC is a challenge. We have started educational initiatives within our clinical and research areas to address this. These initiatives not only enable us to disseminate information about the warehouse and promote adoption across the institution, they also provide a feedback mechanism for additional use cases. 

### 4.2. Importance of Metadata

Information specific to the specimen from which genomic results were derived is very important for making actionable decisions. In addition to universal metadata elements such as identifiers, elements like anatomical source, specimen tissue type, and specimen quality measures can significantly increase the value of genomic results. 

A patient may have both tumor (diseased sample) and germ line (normal sample) sequenced. Without the metadata to help distinguish these samples from each other and identifying the type of tumor, the genomic data may not be as useful or even be considered useless to many studies. For example, the same tumor tissue might be irrelevant to a particular research question or could provide insight into potential targeted treatments or more precise diagnosis in clinical care settings [2]. We have discovered that not all required information with the sample are readily available for all studies, so we are encouraging the inclusion of robust metadata in the study design in order to maximize the value of each genome.

### 4.3. Result Revisions

Re-sequencing of the same sample, reanalysis of the same sequencing results, and re-loading of the same analysis result files can all produce updated versions of a given genome within the warehouse. Should the revised results replace the originals, or should both versions be retained? It depends on the specific circumstances. We found that having multiple versions of same results can cause problems in certain queries, including double or triple counting of samples with multiple versions. 

The presence of multiple versions also adds unnecessary complexity to the retrieval of only the most recent version. However, removing the old results from large tables in the warehouse turned out to be quite time consuming and heavily taxing on the system resource. 

Should the revised results be kept or removed? The decision is multi-facet depending on the type of revision and if it is a clinical or research sample. For clinical samples, the decision was more straightforward—delete them. For research samples, the first type of revision results are retained because technically it is a different experiment that may have different genomic coverage, the second and third types of old revision results should be removed as they are corrected by the new revision results. Keeping previous revisions could be a challenge depending on how specimens undergoing genetic testing are identified and labeled. We found the key to managing this process is to define the lab workflow and document our policy and process for loading as precisely as possible.

### 4.4. Balance Clinical and Research Needs

The Mayo Clinic’s unique structure requires CIM to balance research and clinical needs for data, and these needs sometimes diverge. Researchers on the cutting edge of individualized medicine want frequent upgrades to systems as new techniques are developed, and they also need flexibility to implement new uses as they are developed. In contrast, clinical laboratories are highly regulated and require CLIA (Clinical Laboratory Improvement Act) compliance with an emphasis on validation; in addition, clinical workflows are generally intolerant of downtime and therefore redundancy and high availability must be built into the system or at least planned for when test volumes increase in the future. 

The integrated presence of research and clinical uses also necessitates a barrier between research and clinical data to protect patient privacy and comply with the HIPAA and other regulations

### 4.5. Clinical Samples

TRC stores both phenotypic and genotypic data from patients seen in Mayo’s various clinical environments. The clinical phenotypic data includes: demographic information, diagnosis, procedures, observations (including laboratory results), and medications. An additional phenotypic data type that is possible to load but not yet loaded at Mayo Clinic is patient encounters. The loading of clinical phenotype data started in February 2013 followed by clinical samples in November 2014, and clinical genomic result files (in VCF format) in January 2015.

Currently clinical samples are sequenced for a set of genes (gene panel test) and thus genomic data produced are much smaller in data volume compared to research samples, which are typically sequenced for the entire exome. Indicators for the type of genetic test conducted are stored in the warehouse and can be used in queries to identify variants detected specific to a test or panel. The ability to query genomic test metadata is limited to direct query access to the warehouse currently. In the clinical setting, issues such as reimbursement and ethical concerns regarding incidental findings need to be resolved before whole-exome sequencing will become common use. However, Mayo is committed in bringing whole-exome sequencing to the clinical practice.

Nonetheless, the clinical information is crucial to the utility of the TRC warehouse. As described in the Biobank use case in Section 3.1.1, the availability of the clinical samples from Biobank participants with rich clinical data and genomic results allows researchers to perform feasibility analyses for potential new research studies very quickly. Storing clinical genomic results with associated patient phenotypes can equip a practice with the foundation necessary to store knowledge derived from the science in a way that can be enhanced for future advancement and automation. Specifically, harnessing the experience gained from clinical interpretation of genetic mutation and how it relates to clinical presentation has tremendous potential to streamline this area and allow for automation with machine learning based algorithms.

### 4.6. Future Directions

Going forward, we will work with the end users to develop additional applications to meet their needs and increase the pace of discovery and translation to clinical practice. The plan for 2017 includes the development of APIs to build a suite of new applications to support existing and emerging clinical and research workflows as well as the support for structured variants, copy number variation, proteomic, epigenomic, metabolomic and microbiomic data.

Our priorities are driven in part by the end user, in that data types with strong proponents for inclusion will be the first to be implemented, if we have the technical capability to do so. Managing expectations for delivery of new capabilities can be challenging due to typical release cycle of major updates to TRC from Oracle per year. By being transparent with our stakeholders and end users about our time lines and limitations, we hope to match demand to the delivery of new functionality as close as possible.

Eventually the costs of sequencing and informatics processing to transform raw sequence data will fall to a level that makes whole-genome assays affordable at a population level. TRC will need to be positioned to provide the scalability and capacity to ingest, store and query this massive amount of information.

In the subsequent phases, we will focus on developing a new set of applications surrounding TRC and adding support for additional data types as required by the practice and research. Besides investing in advanced hardware and software, additional investment will be required in the scientific and IT resources to operate the system and to translate complex medical questions through visualization tools and other information delivery mechanisms within our health care IT landscape.

### 4.7. Summary

Challenges exist with an undertaking such as this as our experience shows. Fortunately, we have found that these challenges can be met by focusing on communication and engagement of stakeholders. Prioritization of stakeholder requirements and focus on data acquisition can help an institution gain momentum through implementation and set the pace for driving value from the system.

## 5. Conclusions 

Implementing TRC at Mayo was a strategic decision driven by the desire of the Mayo Clinic to be a leader in individualized medicine. By providing access to the integrated genomic data and clinical data, TRC is poised to play a critical role in advancing both research and clinical care at the Mayo Clinic. Continued growth of this genomic warehouse will provide greater statistical power to support clinical diagnoses and treatment decisions and to turbocharge the discovery and translation to clinical practice in Mayo Clinic.

Collaboration across organizational boundaries and across multiple disciplines was essential. Early engagement with the end users has proven to be critical to the success of the project. As this genomic ecosystem grows and evolves, it will continue to push the capabilities of our research and translational programs forward to advance care for Mayo Clinic patients. 

## Figures and Tables

**Table 1:** Mayo Oracle Translational Research Center (TRC) implementation resources.

Area	Role	Number of Members
IT	Database Administrator	2
IT	Data Pipeline Architect	2
IT	Architect	2
IT	Programmer	6
IT	Support Analyst	2
Bioinformatics	Bioinformatician	2
Biostatistics	Data Scientist	2
Project Management	Project Manager	2
Executive	IT Executive	2
Executive	Clinician	1

IT: Information Technology.

**Table 2:** Mayo Oracle TRC production hardware.

Component	Quantity	CPU	Memory	Disk Space	Manufacturer
Oracle Exadata Database	2	Intel Xeon X5675 24 Core	192 GB	19 TB	Oracle, Redwood City, CA, USA
Application Server	2	Intel Xeon X5687 16 Core	24 GB	500 GB	Hewlett-Packard, Palo Alto, CA, USA
Oracle ZFS Storage Appliance	1	N/A	N/A	2.5 TB	Oracle, Redwood City, CA, USA

**Table 3:** Mayo Oracle TRC reference data.

Data Set	Version
Cosmic	V75
Ensembl	ENS.76.GRCH38, ENS.73.GRCH37
HUGO Gene Nomenclature Committee (HGNC)	2015_10
Human Genome Mutation Database (HGMD)	2015.3
PathwayCommons	2013_09
PolyPhen	GRCH38, GRCH37.P12
Sorting Intolerant From Tolerant SIFT	GRCH38, GRCH37.P12
SwissProt	2015_11, 2013_09

**Table 4:** Mayo Oracle TRC post-implementation resources.

Area	Role	Number of Members
IT	Database Administrator	1
IT	Architect	1
IT	Programmer	2
IT	Support Analyst	2
Bioinformatics	Bioinformatician	As-needed
Project Management	Project Manager	1

**Table 5:** Mayo Clinic genomic data warehouse data statistics.

Data Type	Total
Samples with Genomic Results	11,734
Research Samples	9712
Clinical Samples	2022
Research Studies with Genomic Results	71
Total Variant Count	8,612,759,579
Total Omics Results (Rows)	68,431,547,534
Total Patient Count	9,283,510
Total Subject Count	149,714

## Abbreviations and Acronyms

**Abbreviation/Acronym**	**Definition**
NGS	Next-Gen Sequencing
VCF	Variant Call Format
gVCF	Genomic Variant Call Format
CIM	Center for Individualized Medicine
PGx	Pharmacogenomics
IM Clinic	Individualized Medicine Clinic
EHR	Electronic Health Record
TRC	Translational Research Center
UDP	Unified Data Platform
EDT	Enterprise Data Trust
BORA	Biologically Oriented Repository Architecture
SNP	Single Nucleotide Polymorphism
SQL	Structured Query Language
MVS	Mayo Variant Summary
API	Application Programming Interface
LIMS	Lab Information Management System
WFM	Workflow Manager
ODI	Oracle Data Integrator
ZFS	Zettabyte File System
ETL	Extract, Transform, and Load
HPC	High-Performance Computing
DNA	Deoxyribonucleic Acid
RNA	Ribo Nucleic Acid
IRB	Institutional Review Board
VPD	Virtual Private Database
CLIA	Clinical Laboratory Improvement Act
HIPAA	Healthcare Improvement Portability and Accountability Act
